# Reduced bonobo MHC class I diversity predicts a reduced viral peptide binding ability compared to chimpanzees

**DOI:** 10.1186/s12862-019-1352-0

**Published:** 2019-01-10

**Authors:** Vincent Maibach, Linda Vigilant

**Affiliations:** Department of Primatology, Max Planck Institute for Evolutionary Anthropology, Deutscher Platz 6, 04103 Leipzig, Germany

**Keywords:** *Pan paniscus*, *Pan troglodytes*, Great apes, NetMHCpan, Major histocompatibility complex

## Abstract

**Background:**

The highly polymorphic genes of the major histocompatibility complex (MHC) class I are involved in defense against viruses and other intracellular pathogens. Although several studies found reduced MHC class I diversity in bonobos in comparison to the closely related chimpanzee, it is unclear if this lower diversity also influences the functional ability of MHC class I molecules in bonobos. Here, we use a bioinformatic approach to analyze the viral peptide binding ability of all published bonobo MHC class I molecules (*n* = 58) in comparison to all published chimpanzee MHC class I molecules (*n* = 161) for the class I loci *A*, *B*, *C* and *A-like*.

**Results:**

We examined the peptide binding ability of all 219 different MHC class I molecules to 5,788,712 peptides derived from 1432 different primate viruses and analyzed the percentage of bound peptides and the overlap of the peptide binding repertoires of the two species. We conducted multiple levels of analysis on the “species”-, “population”- and “individual”-level to account for the characterization of MHC variation in a larger number of chimpanzees and their broader geographic distribution. We found a lower percentage of bound peptides in bonobos at the *B* locus in the “population”-level comparison and at the *B* and *C* loci in the “individual”-level comparison. Furthermore, we found evidence of a limited peptide binding repertoire in bonobos by tree-based visualization of functional clustering of MHC molecules, as well as an analysis of peptides bound by both species.

**Conclusion:**

Our results suggest a reduced MHC class I viral peptide binding ability at the *B* and *C* loci in bonobos compared to chimpanzees. The effects of this finding on the immune defense against viruses in wild living bonobos are unclear. However, special caution is needed to prevent introduction and spread of new viruses to bonobos, as their defensive ability to cope with new viruses could be limited compared to chimpanzees.

**Electronic supplementary material:**

The online version of this article (10.1186/s12862-019-1352-0) contains supplementary material, which is available to authorized users.

## Background

The most diverse genes found in higher vertebrates constitute the major histocompatibility complex (MHC). These genes underpin the mammalian adaptive immune system by coding for molecules presenting peptides to immunocompetent cells [1–3]. MHC molecules comprise two main classes: MHC class I molecules present peptides derived from intracellular pathogens while MHC class II molecules present peptides from the extracellular environment, including peptides from extracellular pathogens (reviewed in [4, 5]). The interaction of MHC molecules with immunocompetent cells triggers an immune response. In addition to their role in the adaptive immune system, MHC class I molecules are also involved in the innate immune system by interacting with the killer cell immunoglobulin-like receptors (KIR receptors) occurring on natural killer cells (NK cells) and controlling the NK cell response, which results in lysis of the infected cells [6, 7].

The classical MHC class I genes are defined in humans as *HLA-A*, -*B* and -*C* and orthologs are found in chimpanzees (*Pan troglodytes*), bonobos (*Pan paniscus*) and gorillas (*Gorilla gorilla* and *Gorilla beringei*) [8]. Chimpanzees and bonobos are the closest evolutionary relatives of humans with an estimated most recent common ancestor between 1 and 2.6 mya ([9, 10], reviewed in [11]). Despite the recent divergence of bonobos and chimpanzees, the latter species possesses an additional class I locus, termed *A-like*, which is not present in any other African ape species [12, 13]. Chimpanzees are classified as four geographically delimited subspecies, which have differing population histories and average genetic heterozygosities [14–17]. In contrast, bonobos exist in a single geographic range comparable in size to the range of a single chimpanzee subspecies and have limited genetic substructure [18, 19].

The high variability of the MHC genes is thought to be due to an evolutionary “arms race” between pathogens and the immune system which results in a dynamic process affecting frequencies of MHC alleles and pathogens [20–23]. Nonetheless, diversity in the MHC genes can be maintained over long evolutionary time spans, resulting in balanced polymorphism, which in turn could explain the evolution of MHC “supertypes” [24, 25]. The evolutionary forces and selective pressures responsible for shaping the evolutionary histories of MHC class I loci could result in differences in relative diversity of MHC alleles, as well as in differences in the presence or absence of MHC alleles and lineages in related species. Studies of the bonobo MHC class I loci indicated a lower nucleotide diversity at the *B* locus compared to three of the four chimpanzee subspecies, namely central chimpanzees (*P. t. troglodytes*), eastern chimpanzees (*P. t. schweinfurthii*) and western chimpanzees (*P. t. verus*) [26, 27]. Furthermore, bonobos had significantly less nucleotide diversity than central chimpanzees at the *A* and *C* loci [26]. In addition to reduced nucleotide diversity, a comparison of intron 2 lineages of the *A*, *B* and *C* loci showed that the bonobo intron 2 lineage repertoire is diminished in comparison to chimpanzees [28]. This low MHC diversity in bonobos may be due to a bottleneck, founder effect or selective processes mediated by pathogens after the split from chimpanzees [26–28].

Although assessment of DNA sequence diversity at MHC loci is common in wildlife studies [29–33], it is not clear whether reduced MHC diversity, such as might occur in endangered taxa due to bottlenecks or inbreeding, is associated with reduced population viability [30]. In addition, geographically disparate populations of the same species may experience different selective pressures from exposure to different types or intensities of parasites, further complicating attempts to make inferences by comparing levels of MHC variation among populations. Perhaps most importantly, the extent to which diversity levels reflect immune functionality is not clear [30].

One way to examine the relationship between MHC sequences and immune functionality is through investigation of binding affinities of the MHC molecules. Traditionally, painstaking experimentation has been necessary to investigate the binding affinities of MHC molecules to particular peptides, and the number of MHC molecules and peptides that has been investigated is accordingly limited [34, 35]. For example, although more than 8000 human class I MHC molecules are known (IPD 2017, [36]), fewer than 5% have been subject to experimental assessment of binding affinities (IEDB 2017 [37]). This has motivated the development of bioinformatic tools to predict binding affinities of MHC molecules and peptides, with a particular focus on the class I *A* and *B* loci in humans [38]. Recent extension of these approaches to encompass the class I *A*, *B*, and *C* loci and incorporate information from non-human taxa provides the opportunity to assess the immune response, via the proxy of estimated peptide-binding ability, in individual representatives of wildlife populations [39].

Here we use the peptide binding prediction tool *NetMHCpan* [38, 40] to assess the binding affinities of all known MHC class I molecules of the bonobo *Papa-A*, *Papa-B*, *Papa-C* loci and of the chimpanzee *Patr-A*, *Patr-B*, *Patr-C* and *Patr-A-like* loci, respectively. To accomplish this we use peptides derived from a biologically relevant set of more than 1400 different primate viruses (Virus host DB, [41]). Given previous evidence of a reduced MHC class I diversity in bonobos compared to chimpanzees [26–28] as well as the lack of the *A-like* locus in bonobos, we hypothesize that bonobos also have a limited capacity to bind viral peptides as compared to chimpanzees. To assess our hypothesis, we analyze the binding prediction data from several different perspectives. First, we compare the number or the percentage of bound peptides using a) all known bonobo and chimpanzee MHC class I alleles (“species”-level), b) equal samples of central chimpanzees, western chimpanzees and bonobos (“population”-level), c) 20 bonobo, 20 central chimpanzee and 20 western chimpanzee samples individually (“individual”-level). Second, we examine the overlap in the peptide binding repertoires of the two species using a) functional trees produced by *MHCcluster* and b) a comparison of the proportion of peptides bound by both bonobos and chimpanzees (“shared peptides”) by including all known MHC class I alleles (“species”-level) and only MHC class I alleles found in a sample of 20 bonobos, 20 central chimpanzees and 20 western chimpanzees (“population”-level).

## Methods

### MHC – Class I binding prediction

We used *NetMHCpan* 3.0, a bioinformatic tool using artificial neutral networks to predict binding specifies of peptides to MHC class I molecules [38, 40]. Training of the artificial neural networks with nonhuman MHC sequences and experimentally determined affinity data makes this software a useful tool for the prediction of binding affinities in chimpanzees and other species [39, 42]. We included all available chimpanzee and bonobo MHC class I protein sequences retrieved from the IPD-MHC NHP database [43] (date June 2017) for analysis. In total we used 219 MHC class I protein sequences including 20, 27, 11, 41, 78, 38 and 4 different sequences for the different loci *Papa-A*, *Papa-B*, *Papa-C*, *Patr-A*, *Patr-B*, *Patr-C* and *Patr-A-like*, respectively (for details see Additional file 1).

**Fig. 1:**
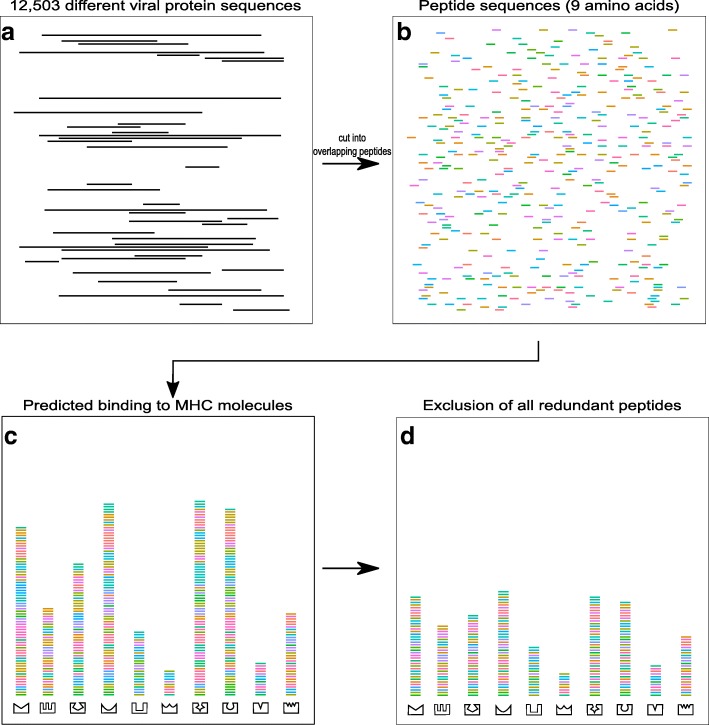
Schematic flowchart of the data production. **a** A total of 12,503 different viral protein sequences representing 1432 different viruses known to infect primates were downloaded from Virus-host DB [41] **b**. The viral protein sequences were cut into small (nine amino acid) overlapping peptides by *NetMHCpan*, resulting in 5,788,712 peptide sequences. **c** We used *NetMHCpan* to predict the binding abilities of 219 different bonobo and chimpanzees MHC class I molecules for the loci *A*, *B*, *C* and *A-like* to all viral peptides in the dataset. Cartoons of different MHC molecules and their predicted peptide binding repertoire are shown at the bottom of the figure. **d** For each MHC molecule we removed all redundant peptides, e.g. for peptides which were bound more than once at a particular MHC molecule, we kept only one representative peptide. In total, our dataset contained 2,791,353 peptide sequences, each with a unique amino acid sequence

For the creation of our viral peptide dataset we used data from Virus-host DB [41], a database which contains complete genomes stored either in NCBI/RefSeq [44] or EBI Genomes [45] and respective host information. We downloaded all protein sequences of all viruses known to infect primates, resulting in 12,503 different proteins sequences derived from 1432 different viruses. We decided to use all primate viruses rather than viruses only affecting chimpanzees and bonobos both because many viruses may infect a range of species, as well as due to the lack of host information for those two species, which would have led to an insufficient number of viruses for this study. Protein sequences were digested by *NetMHCpan* into small overlapping peptides with a length of nine amino acids because MHC class I molecules preferentially bind peptides of nine amino acids in length [40]. The digestion of the 12,503 different proteins sequences resulted in 5,788,712 peptides, of which 2,791,353 had a unique peptide sequence (Fig. 1). The binding affinities of all peptide sequences to all 219 different MHC alleles were predicted using *NetMHCpan*. We defined a peptide to be bound by a MHC molecule if the predicted binding affinity was below 500 nanomolar (nM), because a low value in binding affinity represents a stronger bond between two molecules [46]. We chose to use a defined affinity threshold rather than percentile rank scores or a specific percentage threshold in order to avoid the assumption that all MHC molecules bind the same number of peptides. Experimental data also supports the use of a defined affinity threshold with a fixed value of 500 nM, but we recognize that the number of bound peptides could nevertheless be over- or underestimated for some MHC molecules [47].

We next excluded all redundant peptides for each MHC molecule, e.g. we kept only one representative peptide for each peptide which occurred with the same amino acid sequence multiple times. The exclusion of all redundant peptides might lead to a loss of information, because highly redundant peptides could indicate very important peptides, which could be prioritized by MHC molecules. We therefore tested if the number of occurrences of peptides correlates with the number of bound MHC molecules. We further define “shared peptides” as those which could be bound by both bonobo and chimpanzee MHC molecules. In this study we use the term binding repertoire or peptide binding repertoire to describe the total set of peptides which could be bound by the individual MHC molecules. The sum of binding repertoires of the individual MHC molecules from one species defines the peptide binding repertoire of the whole species.

### MHCcluster

*MHCcluster* 2.0 graphically depicts the predicted functionality of the MHC molecules by clustering MHC molecules based on their predicted binding specificities derived using *NetMHCpan* (version 2.7) [48]. *MHCcluster* uses a predefined set of natural peptides to predict binding specificities of each MHC molecule, compares the similarities of those specificities to each other MHC molecule, transfers the similarities to a distance matrix and, finally, converts the distance matrix into an unrooted UPGMA tree [48]. We used R (version 3.1.3) with the package ape (version 3.4) and Inkscape (version 0.92) to graphically modify the UPGMA trees.

### Data analysis

We used the data from *NetMHCpan* and *MHCcluster* to compare the viral peptide binding capabilities of bonobo and chimpanzee MHC class I molecules from two different perspectives. First, we compared the number or percentage of different viral peptides bound by bonobo or chimpanzee MHC molecules and compared these according to species, population and individual. Second, we analyzed the overlap of the binding repertoires of the two species using both the functional trees produced by *MHCcluster* and the analysis of the peptides bound by both species using the *NetMHCpan* data.

### Comparison of percentage of bound viral peptides

We defined for each MHC molecule the percentage of bound peptides (e.g. number of peptides bound by the MHC molecule multiplied by 100 and divided by the total number of peptides in the dataset with a unique peptide sequence) and compared them between the two species. To fully describe all potential differences between the two species we analyzed the data on three different levels referring to them as the “species”-, “population”- and “individual”-level. For the “species”-level we used all known MHC molecules for the comparison, grouped all peptides from each MHC molecule for each locus and species, defined from that the peptides per locus and species and calculated the percentage of peptides, e.g. creating for each locus and for each species one value, whereby counting peptides bound by multiple MHC molecules within one locus and species only once. Considering the notable difference in the number of known MHC molecules between the two species (58 vs. 157 for the *A*, *B* and *C* loci together for bonobos and chimpanzees, respectively) we analyzed the peptide binding data on the “population”- and “individual”-level to account for the larger and potentially broader geographic sampling of MHC molecules in chimpanzees. In a recent study we genotyped 20 different unrelated bonobos and 20 central chimpanzees for the MHC loci *A*, *B* and *C* (see Additional file 2) [26]. Based on this genotype information and genotype information from 20 western chimpanzees [49] we created a subset from the whole dataset, comprising only binding predictions for MHC molecules from those 20 bonobos, 20 central chimpanzees and 20 western chimpanzees. We refer to use of this subset as a comparison on the “population”-level. Sufficient information for the other two chimpanzee subspecies, the eastern chimpanzees and the Nigeria-Cameroon chimpanzees (*Pan troglodytes ellioti*) were not available during the course of this study and are therefore not included in our comparisons. Again, we calculated for each locus and each species/subspecies the percentage of different peptides as described for the “species”-level comparison. The binding prediction for one MHC molecule (Patr-C*01:01) for one individual from our western chimpanzee “population” was not possible, due to missing sequence information in the exon 2 of this particular MHC molecule. For the “individual”-level comparison we used again the genotype information of the 20 bonobos, 20 central chimpanzees and 20 western chimpanzees and calculated for each individual the percentage of bound peptides for each of the three loci *A*, *B* and *C* separately (e.g. one value for each loci and individual; peptides bound by multiple MHC molecules within one locus and species were not excluded).

### Overlap of the binding repertoires of the two species

We used two approaches to analyze the overlap of the binding repertoires of the two species. First, we produced for each of the loci *A*, *B* and *C* a functional tree with *MHCcluster*, whereby molecules for the *A-like* locus were included in the *A* tree based on their similarity to the *A* locus [12, 13]. Second, we estimated the percentage of peptides of the *NetMHCpan* data bound by both species (“shared peptides”). For this we calculated for each locus and species the total number of bound peptides and identified those peptides which were bound by both bonobos and chimpanzees. Furthermore, we calculated the percentage of peptides which were not bound by the other species (“species specific peptides”). This was done for the “species”-level comparison and for the “population”-level (20 bonobos, 20 central chimpanzees and 20 western chimpanzees) comparison.

### Statistics

We conducted a permutation test (10,000 permutations) for the “population”-level comparison of the percentage of bound peptides between the two species/subspecies for each locus [50]. We grouped all peptide data of the two groups of the particular comparison (e.g. 20 bonobos, 20 central chimpanzees or 20 western chimpanzees for one of the three loci *A*, *B* and *C*). For each permutation, we resampled for each group 20 individuals independent of their species origin with their corresponding peptide data, calculated the number of peptides for each group and calculated the absolute difference of this value between the two groups. We compared the absolute differences of each permutation with the absolute difference of the original groups. The proportion of absolute differences of every permutation greater than or equal to the difference of the two original groups was defined as the *p*-value.

For the statistical comparison at the “individual”-level, we used a Welch’s t-test. This test was preferred over a Wilcoxon-Mann-Whitney-Test (U-test) because the variances of the samples were unequal. We corrected all *p*-values of all statistical tests for multiple testing with the Bonferroni correction if necessary. All statistical analyses were executed in R (version 3.1.3).

## Results

In total we bioinformatically compared 219 different MHC molecules to a set of 5,788,712 nine amino acid long viral peptides derived from a dataset of 1432 viruses. The individual MHC molecules bound, in silico, between 0 and 239,570 viral peptides each (Additional file 1). Four MHC molecules (Papa-B*13:01, Patr-B*08:01, Patr-B*08:02 and Patr-B*21:02) bound no peptides below the affinity threshold of 500 nM. Although some peptides occur up to 243 times in our dataset, these represent a very small fraction of the total dataset. For example, peptides occurring six or more times represent 3.9% of the data while the majority of the peptides occur only once and represent 69.9% of the data (Additional file 3). We found no correlation between the number of occurrences of a peptide in our dataset and the number of MHC molecules which bound those peptides, suggesting that even some common peptides sequences may not be bound by multiple MHC molecules (Pearson’s product-moment correlation, *t* = − 2.4937, df = 108,740, *p* = 0.01264, *r* = − 0.0076). Furthermore, we found a strong correlation between the number of bound common peptides (peptides that occurred six or more times in our data set) and the number of total bound peptides for each MHC molecule (Pearson’s product-moment correlation *t* = 389.66, df = 210, p = < 0.00001, *r* = 0.9993). This implies that if a MHC molecule bound a large number of common peptides it also bound a large number of total peptides overall and vice versa, a low number of total bound peptides resulted in a low number of bound common peptides. In addition, we calculate for each MHC molecule the percentage of bound common peptides based on their number of total bound peptides. The percentages were comparable between the different MHC molecules and the average percentage for all MHC molecules of 3.9% was identical to the percentage of peptides in our dataset occurring six or more times. In consequence, this implies that there were no special MHC molecules binding common peptide sequences.

### Comparison of percentage of bound viral peptides

**Fig. 2:**
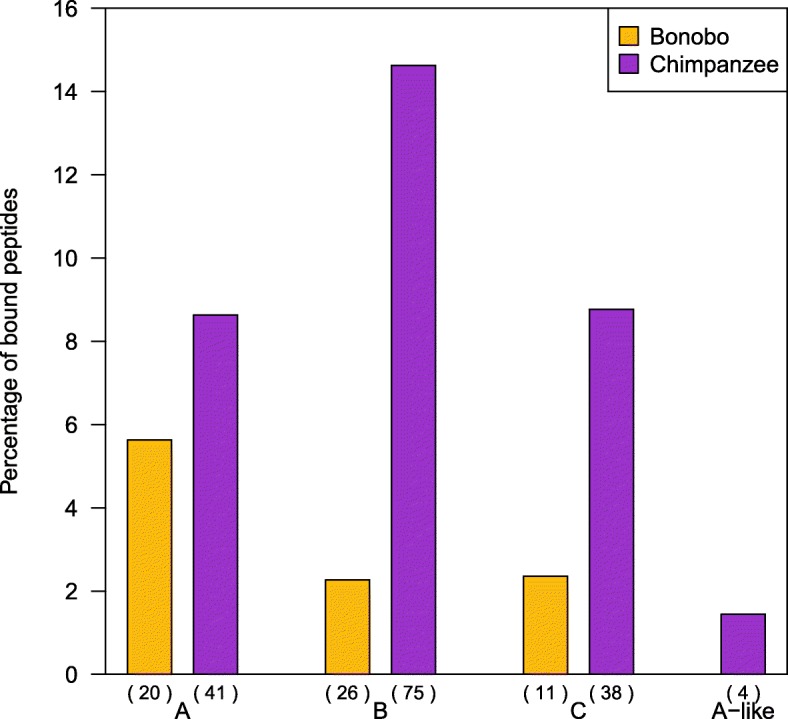
“Species”-level comparison of bound peptides. The percentage of bound peptides for bonobos and chimpanzees including all published MHC class I molecules for the *A*, *B*, *C* and *A-like* loci. Peptides bound by multiple MHC molecules within one locus and species were counted only once. The different loci and the number of MHC molecules for each species and loci in this comparison are indicated below each corresponding bar

**Fig. 3:**
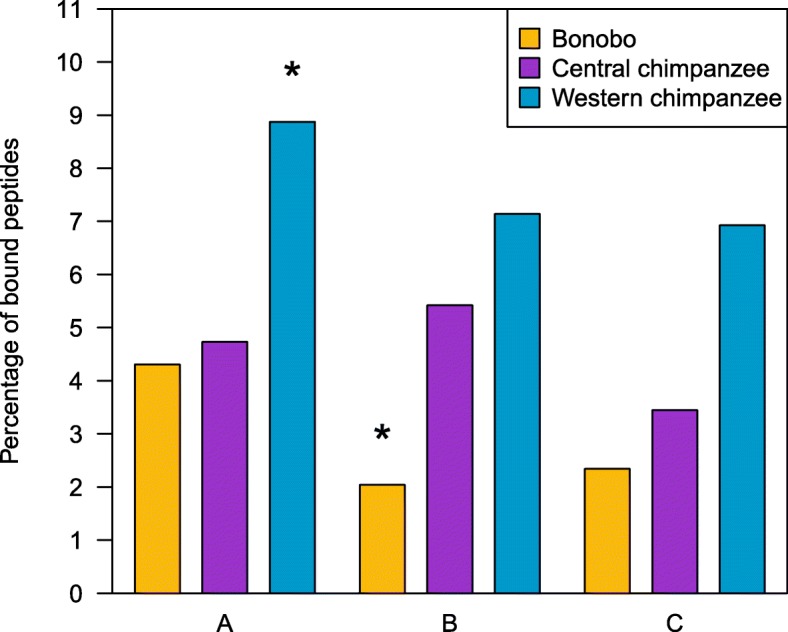
“Population”-level comparison of bound peptides. The percentage of bound peptides for 20 bonobos, 20 central chimpanzees and 20 western chimpanzees for the *A*, *B* and *C* loci. Peptides bound by multiple MHC molecules within one locus and species/subspecies were counted only once. Significant differences between the different species/subspecies within each locus are indicated by stars above the different bars, thereby a star above a bar represents a significant difference to each of the other two bars within this locus (permutation test, 10,000 resamplings, Bonferroni correction for multiple testing, *p* ≤ 0.0052 significant threshold after Bonferroni correction)

A comparison of the range, the average number and the percentage of predicted bound peptides for each MHC locus suggests that, when using all reported -*A*, -*B* and -*C* molecules from the two species, our sample of bonobos binds approximately four- to seven-times fewer peptides than the sample of chimpanzees at the *B* and *C* loci (Table 1 and Fig. 2). However, the apparent differences may be influenced by the characterization of MHC variation from a larger number of chimpanzees, possibly of widespread geographic origin, relative to bonobos. Therefore, we next compared the proportion of predicted bound peptides for sets of 20 individuals from bonobos, central chimpanzees and western chimpanzees, respectively [26, 49]. In this “population”-level comparison (Fig. 3) bonobos showed a significantly lower percentage of bound peptides than central chimpanzees and western chimpanzees at the *B* locus (permutation test, Bonferroni correction, *p* = 0.0018 and *p* = 0.0001, respectively), whereas western chimpanzees exhibited a greater percentage of bound peptides than bonobos and central chimpanzees at the *A* locus (permutation test, Bonferroni correction, *p* = 0.0021 and *p* = 0.0052, respectively). There were no significant differences at the *C* locus (permutation test, Bonferroni correction, *p* ≥ 0.0056) (Additional file 4).

**Table 1:** Number and percentage of predicted bound peptides per MHC molecule for the different bonobo (*Papa*-) and chimpanzee (*Patr*-) MHC class I loci

Locus	*Papa-A*	*Papa-B*	*Papa-C*	*Patr-A*	*Patr-B*	*Patr-C*	*Patr-A-like*
No. of MHC molecules	20	27	11	41	78	38	4
Predicted bound peptides							
minimum	458	0	618	51	0	620	40,365
maximum	80,337	34,689	40,976	104,816	239,570	181,572	40,365
average	29,657	3834	10,499	27,413	17,642	33,172	40,365
Percentage of average	5.63	2.27	2.36	8.63	14.63	8.77	1.45

The number of MHC molecules comprises the number of MHC molecules used in this study for each locus, which are all reported bonobo and chimpanzee MHC class I molecules

**Fig. 4:**
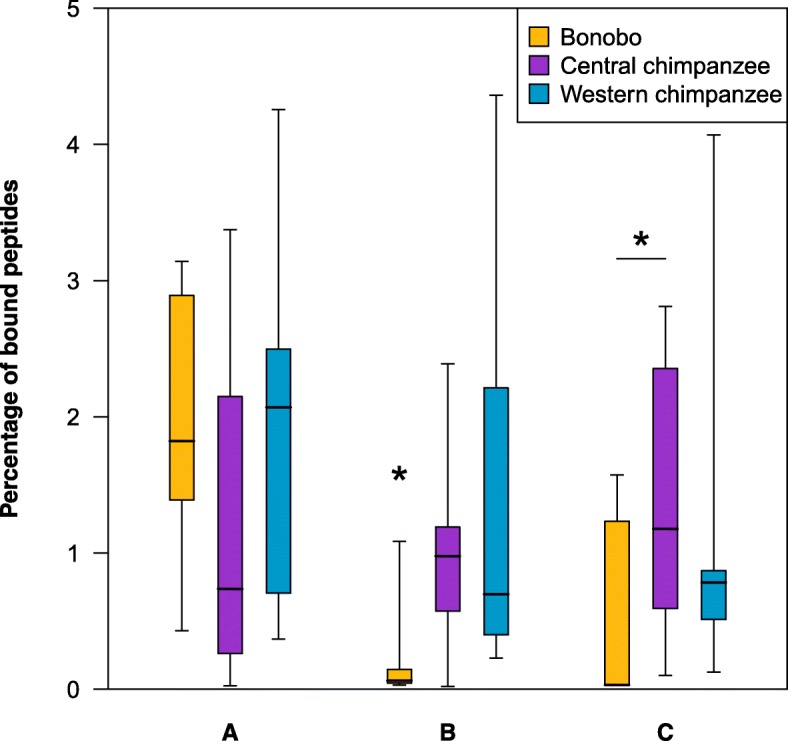
“Individual”-level comparison of bound peptides. The percentage of bound peptides for 20 individual bonobos, 20 individual central chimpanzees and 20 individual western chimpanzees for the *A*, *B* and *C* loci. From top to bottom the whiskers and the box of each boxplot represents the 2.5th, 25th, 50th (median), 75th and 97.5th percentiles. Peptides bound by multiple MHC molecules within each individual for each loci and species/subspecies were counted only once. Significant differences between the different species/subspecies within each locus are indicated by stars above the different bars, thereby big stars above a bar represents significant differences to the other two bars within the corresponding locus and small stars with lines represents a significant difference between the two connected bars (Welch’s t-test, Bonferroni correction for multiple testing, *p* ≤ 0.00016 significant threshold after Bonferroni correction)

We next tested if the differences between bonobos and central chimpanzees in the “population”-level comparison were also present at the individual level. In contrast to the “population”-level comparison, this analysis estimated for each individual at each locus a proportion of bound peptides. As in the “population”-level comparison, we found that at the *B* locus, individual bonobos bound a significantly lower proportion of peptides than did individual central chimpanzees and individual western chimpanzees (Fig. 4; Welch’s t-test, Bonferroni correction, *t* = − 4.226, df = 36.19, *p* = 0.00015; *t* = − 6.04, df = 36.39, *p* < 0.00001, respectively) (Additional file 4). Furthermore, at the *C* locus individual bonobos also bound significantly fewer peptides than did individual central chimpanzees (Welch’s t-test, Bonferroni correction, *t* = − 4.210, df = 36.59, *p* = 0.00016). Interestingly, in the “individual”-level comparison western chimpanzees did not bind a significantly higher percentage of peptides compared to central chimpanzees and bonobos at the *A* locus, unlike in the “population”-level comparison (Welch’s t-test, Bonferroni correction, *t* = − 1.90, df = 36.90, *p* = 0.06520; *t* = 0.64, df = 37.37, *p* = 0.52307, respectively).

### Overlap of the binding repertoires of the two species

**Fig. 5:**
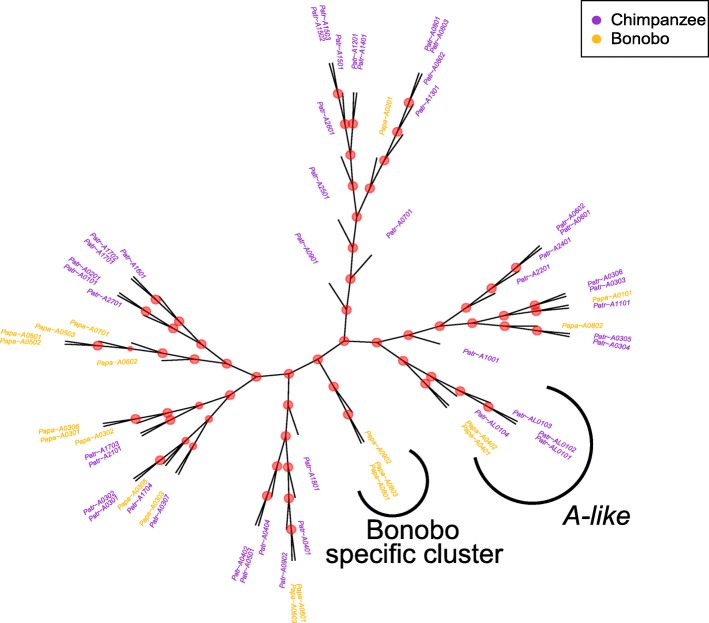
Functional tree of the *A*-locus. Unrooted UPGMA tree showing the functional clustering of all reported *A* molecules for bonobos and chimpanzees, as well as all reported *A-like* molecules for chimpanzees. This tree was produced by using MHCcluster 2.0, R (version 3.1.3) with the package ape (version 3.4) and Inkscape (version 0.92). Close clustering of MHC molecules indicates a similar peptide binding repertoire of the MHC molecules. Bootstrap values at the split points of individual MHC molecules are indicated by red dots, thereby large dots indicate a bootstrap value larger than 0.95, medium dots indicate a bootstrap value between 0.85 and 0.95, and small dots indicate a bootstrap value lower than 0.85. The maximum possible bootstrap value is 1. A bonobo specific cluster and the *A-like* sequences are designated

We next investigated the overlap of the binding repertoires to analyze the extent to which both species bind the same peptides. We used *MHCcluster* to produce UPGMA trees depicting clusters of MHC molecules with similar binding properties. The bonobo MHC-*A* molecules were found in all major chimpanzee clades, (Fig. 5) but conversely in the bonobo cluster of Papa-A*09:02, Papa-A*08:03 and Papa-A*08:01 there was no chimpanzee MHC allele, suggesting a small proportion of bonobo-specific peptide binding properties amid a very similar overall binding repertoire of the two species at this locus. Interestingly, the bonobo MHC molecules Papa-A*04:01 and Papa-A*04:02 clustered together with the molecules from the chimpanzee-specific *A-like* locus. This suggests that even though bonobos as a species do not possess the *A-like* locus, they might possess functionally similar MHC molecules at the *A* locus.

**Fig. 6:**
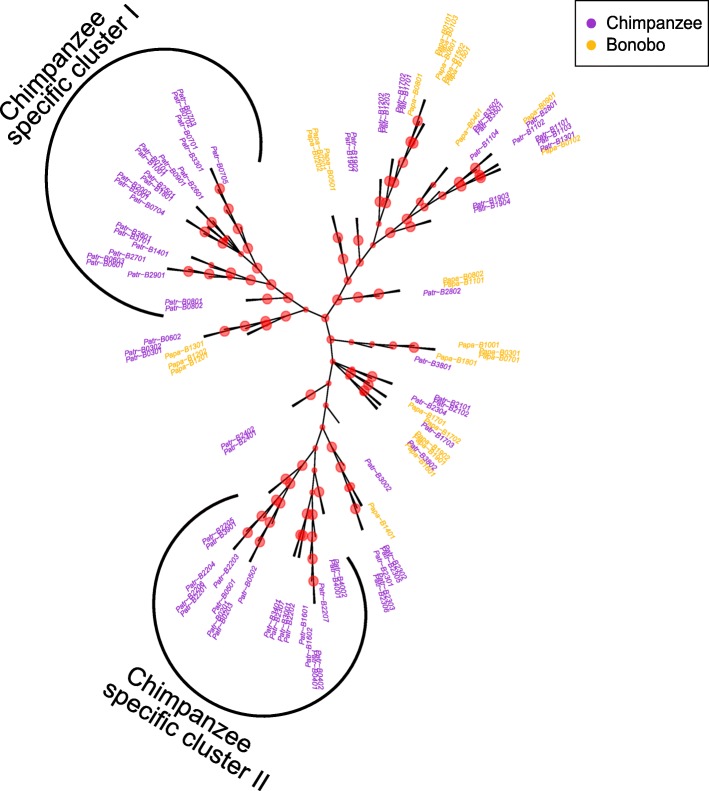
Functional tree of the *B*-locus. Unrooted UPGMA tree showing the functional clustering of all reported *B* molecules for bonobos and chimpanzees. Bootstrap values at the split points of individual MHC molecules are indicated by red dots, thereby large dots indicate a bootstrap value larger than 0.95, medium dots indicate a bootstrap value between 0.85 and 0.95, and small dots indicate a bootstrap value lower than 0.85. The maximum possible bootstrap value is 1. The two chimpanzee clusters with no bonobo MHC molecule sequences are indicated as “chimpanzee specific cluster I” and “chimpanzee specific cluster II”

**Fig. 7:**
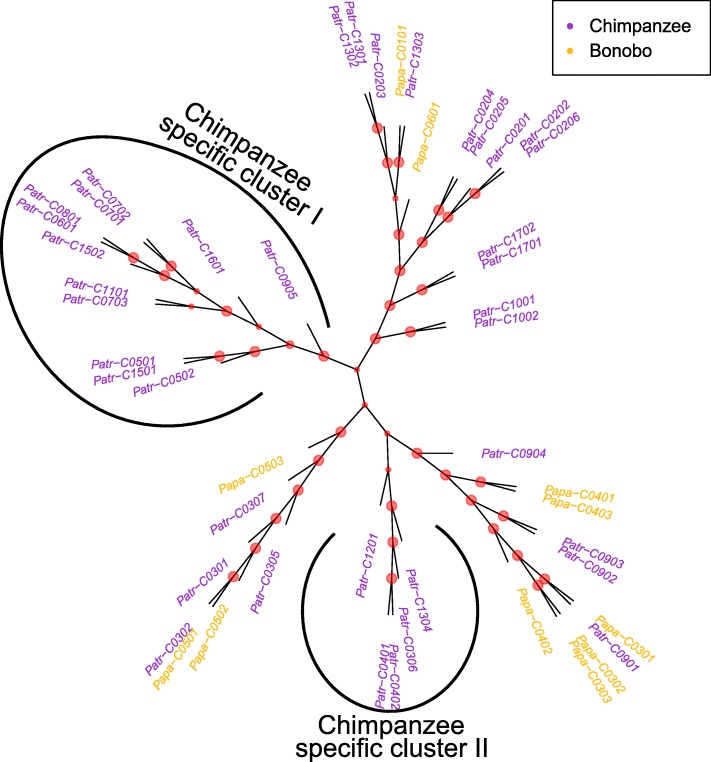
Functional tree of the *C*-locus. Unrooted UPGMA tree showing the functional clustering of all reported *C* molecules for bonobos and chimpanzees. Bootstrap values at the split points of individual MHC molecules are indicated by red dots, thereby large dots indicate a bootstrap value larger than 0.95, medium dots indicate a bootstrap value between 0.85 and 0.95, and small dots indicate a bootstrap value lower than 0.85. The maximum possible bootstrap value is 1. The two chimpanzee clusters with no bonobo MHC molecule sequences are “chimpanzee specific cluster I” and “chimpanzee specific cluster II”

In contrast to the pattern observed for the *A* locus, the functional grouping of MHC-*B* and MHC-*C* molecules differs for the two species (Figs. 6 and 7). Bonobo MHC-*B* as well as MHC-*C* molecules are not represented in two large chimpanzee clades, respectively, suggesting a greater peptide binding repertoire for chimpanzees at both loci. Furthermore, bonobo MHC-*B* and -*C* molecules cluster closer together, whereas chimpanzee molecules appear to be more widespread, indicating that peptides bound by bonobo molecules are more similar than peptides bound by chimpanzee molecules.

After this qualitative analysis of the functional trees, we quantified the observed differences in the binding repertoires of the two species by comparing the proportion of the viral peptide dataset bound by the MHC repertoires of bonobos and chimpanzees, respectively (Table 2). Of the 247,209, 409,677 and 249,775 bound peptides for the three loci, 61.06, 15.13 and 24.39% of them are bound by both species (“shared peptides”), respectively. This indicates a greater overlap in the binding repertoire at the *A* locus than at the *B* or *C* loci, which is in accordance with our inferences from the functional trees. Moreover, the percentages of peptides which are bound only by bonobo MHC molecules are very limited compared to the percentages of peptides bound only by chimpanzee MHC molecules (*A*: 2.51 vs. 36.42, *B*: 0.32 vs. 84.54, *C*: 1.98 vs. 73.63). This means that nearly all peptides bound by bonobos could be also bound by chimpanzees but the majority of chimpanzee-bound peptides at the *B* and *C* loci could not be bound by bonobos.

**Table 2:** “Species”-level comparison of “shared peptides” between bonobos and chimpanzees for the three loci *A*, *B* and *C*

Locus	*A*	*B*	*C*
Total no. bound peptides	247,209	409,677	249,775
Percentage of shared peptides	61.06	15.13	24.39
Percentage of peptides bound only by bonobos	2.51	0.32	1.98
Percentage of peptides bound only by chimpanzees	36.42	84.54	73.63

This comparison includes all reported MHC class I molecules for both species. The total number of bound peptides contains all peptides bound by bonobo MHC molecules and by chimpanzee MHC molecules. We excluded peptides identical to those already included. All percentages in this comparison refer to the total number of bound peptides

To account for the differences in the number of MHC molecules described for bonobos and chimpanzees in the “species”-level comparison of “shared peptides”, we next analyzed the percentages of “shared peptides” with a reduced dataset (“population”-level comparison, e.g. 20 bonobos, 20 central and 20 western chimpanzees). Of the 227,174, 254,270 and 216,409 peptides bound for the *A*, *B* and *C* loci, 38.53, 13.63 and 21.61% of them are bound by all three “populations”, respectively (Table 3). This represents a lower percentage of “shared peptides” at all three loci compared to our “species”-level comparison, with the largest difference at the *A* locus (*A*: 38.53 vs. 61.06, *B*: 13.63 vs. 15.13, *C*: 21.61 vs. 24.39). Comparing the percentages of “shared peptides” between each of the three different “populations”, we found a higher percentage of “shared peptides” between our set of bonobos and our set of western chimpanzees compared to our set of bonobos and our set of central chimpanzees. However, the differences were in each of the three loci relatively small and approximately the same, with the highest difference at the *B* locus with 6.25%. As expected, our set of central and western chimpanzees shared a higher percentage of bound peptides than each of the two to our set of bonobos. Although, the percentages of peptides bound only by bonobos slightly increased compared to the “species”-level comparison at each of the three loci (*A*: from 2.51 to 4.90; *B*: from 0.32 to 1.69; *C*: from 1.98 to 2.89), the percentage of peptides bound only by one of the two sets of chimpanzees is still higher at each of the *A*, *B* and *C* loci (central chimpanzees: 5.82, 19.50, 7.39 and western chimpanzees: 29.13, 32.09, 47.28, respectively). Interestingly, the percentage of peptides bound only by central chimpanzees for the *A* and *C* loci were quite low compared to the percentage of peptides bound only by western chimpanzees (*A*: 5.82 vs. 29.13; *C* 7.39 vs. 47.28).

**Table 3:** “Population”-level comparison of “shared peptides” for a set of 20 bonobos, 20 central chimpanzees and 20 western chimpanzees for the three loci *A*, *B* and *C*

Locus	*A*	*B*	*C*
Total no. of bound peptides	227,174	254,270	216,409
Peptides shared by all three “populations”	38.53%	13.63%	21.61%
Peptides shared by bonobos & central chimpanzees	40.21%	14.07%	21.99%
Peptides shared by bonobos & western chimpanzees	46.34%	20.32%	26.97%
Peptides shared by central & western chimpanzees	50.66%	39.59%	36.69%
Peptides bound only by bonobos	4.90%	1.69%	2.89%
Peptides bound only by central chimpanzees	5.82%	19.50%	7.39%
Peptides bound only by western chimpanzees	29.13%	32.09%	47.28%

The total number of bound peptides contains all peptides bound by the 20 bonobos, 20 central chimpanzees and 20 western chimpanzees. All percentages refer to the total number of bound peptides

## Discussion

Here we used bioinformatic tools to predict the binding capabilities of 219 different bonobo and chimpanzee MHC class I molecules at the *A*, *B, C* and *A-like* loci to peptides derived from over 1400 viruses. When comparing the number of bound peptides using all bonobo and chimpanzee MHC molecule sequences (“species”-level comparison) it appears that bonobo MHC molecules bound fewer peptides than chimpanzees MHC molecules at the *B* and *C* loci. This inference was supported by our “population”- and “individual”-level comparisons, which were conducted to account for the larger characterization of chimpanzee MHC class I molecules, their larger geographic distribution and their higher degree of genetic substructure [16, 18, 19, 51, 52] . When controlling for sample size and chimpanzee subspecies origin, we found that at the “population”-level bonobo MHC class I molecules bound significantly fewer peptides at the *B* locus than did both the western and central chimpanzee subspecies as well as fewer peptides at the *A* locus compared to the western chimpanzee subspecies. In addition, individual bonobos also bound significantly fewer peptides at the *B* locus compared to individual central and western chimpanzees. Furthermore, individual bonobos also bound significantly fewer peptides at the *C* locus than individual central chimpanzees. Thus, the results of the “species”-level comparison and the significant results of the “population”- and “individual”-level comparisons suggest that bonobos have a reduced ability to bind viral peptides in terms of the number of bound peptides compared to chimpanzees. The significant differences between bonobos and chimpanzees were also present even after using a higher binding affinity score of 1000 nM as a threshold for the binding of a peptide by a MHC molecule (data not shown).

The further comparison of the overlap in the peptide binding repertoire between bonobo and chimpanzees MHC class I molecules also clearly suggested a reduced ability of bonobo MHC class I molecules to bind various different peptides compared to chimpanzee MHC class I molecules. Both the absence of bonobo molecules in different chimpanzee clades at the *B* and *C* loci in the functional trees, as well as the drastic difference in the quantified peptide binding repertoires of the two species suggest that chimpanzees MHC class I molecules are able to bind a greater array of different peptides compared to bonobo MHC class I molecules. Chimpanzee MHC class I molecules could bind the vast majority of peptides bound by bonobo MHC class I molecules, however, a large proportion of peptides bound by chimpanzee MHC class I molecules were not bound by bonobo MHC class I molecules. In consequence, this indicates that the whole range of the peptide repertoire bound by bonobo MHC class I molecules is only a subset of the chimpanzee MHC class I peptide repertoire.

Interestingly, in the functional tree for the *A* locus, which included also the *A-like* sequences, we found two bonobo molecules (Papa-A*04:01 and Papa-A*04:02) clustering near the chimpanzee *A-like* molecules, which indicates a similar peptide binding repertoire and therefore functional similarity of these two bonobo molecules. The *A-like* locus, present in about 50% of chimpanzee haplotypes [12], could be of great value in terms of peptide presentation for chimpanzees. This is supported by the high degree of sequence conservation of *A-like* molecules in the antigen binding site, e.g. there are no differences between the known *A-like* molecules in the protein sequences of the antigen binding site, which could be a sign of functional importance of this specific *A-like* sequence [53]. The *A-like* locus in chimpanzees is not the result of a recent duplication event of the chimpanzee *A* locus but rather represents an old duplication of the ancestor of the *A* locus now present in all African ape species [12]. This duplication event likely occurred some 23 million years ago [12, 13] However, the ancient *A-like* locus was lost in the other African ape species, whereby sequence parts of this locus can be found in present day pseudogenes in those species [13]. The ancient duplication of the *A-like* locus and the different evolutionary pathways of this locus in the present day ape species and their ancestors represent an interesting example for the theory of birth and death evolution of MHC genes [54, 55]. The absence of the *A-like* locus in bonobos is puzzling, however the potential disadvantages of lacking the *A-like* locus could have been diminished by maintaining functionally similar *A* molecules. However, it is important to note, that the potential benefit of the *A-like* locus could be exaggerated, as *A-like* molecules have a low expression level as shown in peripheral blood cells and B cells lines [56]. This reduced expression level could influence the peptide binding ability of *A-like* molecules and the true benefit of *A-like* molecules could be rather limited.

In sum, we conclude that bonobo MHC class I molecules have a reduced breadth of viral peptide binding ability as compared to chimpanzee MHC class I molecules. This is in concordance with our hypothesis that the low sequence diversity of bonobo MHC class I genes results in a reduced peptide binding ability at these loci. Low sequence diversity in the different MHC class I alleles as well as the limited number of alleles in bonobos [26–28], leads to relatively similar MHC protein sequences, which in turn would reduce the peptide binding repertoire of bonobo MHC class I molecules. Interestingly, our findings of a reduced peptide binding ability in bonobos at the *B* locus are also supported by a recent study by de Groot et al. [57].

Potential explanations for the reduced sequence diversity and concordant reduced peptide binding ability in bonobos include demographic and/or selective processes. For example, a severe population bottleneck, a founder effect or a selective sweep after the split from chimpanzees were proposed to explain the reduced diversity in bonobo MHC class I genes [26–28]. Concerning the importance of MHC genes to the immune system and potential fitness consequences, selective forces by different pathogens may be the most cogent explanation. For example, the reduced MHC diversity in chimpanzees and bonobos as compared to humans was speculated to be the outcome of a selective sweep mediated by SIV (simian immunodeficiency virus) or another virus in the ancestor of chimpanzee and bonobos [58–61]. Importantly, the different chimpanzee subspecies have different demographic histories and effective population sizes [14–17]. In addition, each chimpanzee subspecies lives in a distinct geographic region within Africa [51], and each subspecies could be confronted with different pathogens leading to different selective pressure on the individual MHC alleles within the different subspecies. Indeed, the chimpanzee form of simian immunodeficiency virus (SIV) has been found only in two of the four chimpanzees subspecies, pointing to different pathogen pressures for the different subspecies (reviewed in [62]). Although we did not focus on subspecies comparisons in chimpanzees in this study, we did find interesting differences between our set of central and western chimpanzees. For example, we did find that western chimpanzees bound significantly more peptides than central chimpanzees at the *A* locus in our “population”-level comparison, but not in our “individual”-level comparison. This suggests, that MHC molecules in our set of central chimpanzees bind more of the same peptides than MHC molecules in our set of western chimpanzees, because same peptides bound by multiple MHC molecules within each locus and species were excluded in the “population”- but not in the “individual”-level comparison. Furthermore, the percentage of peptides bound only to MHC molecules from western chimpanzees were much higher at the *A* and *C* loci compared to the percentage of peptides bound only to MHC molecules from central chimpanzees. This could be an indication of a different pathogen environment of western chimpanzees, as their MHC molecules are able to bind different peptides than central chimpanzees and bonobos. Together, these findings hint at the need in the future to further explore differences among the chimpanzee subspecies. In this regard there is also a need for future studies to compare the MHC class I binding prediction of the two remaining chimpanzee subspecies (eastern and Nigeria-Cameroon chimpanzees) which were not included in our “population”- and “individual”-level comparisons to the MHC class I binding prediction of bonobos.

Conclusions on which virus or viruses could be responsible for the reduced viral peptide binding ability in bonobos or against which viruses chimpanzees might be better protected than bonobos are very difficult to draw from the results of this study and would be very speculative. In an additional analysis (data not shown), we investigated the peptide binding ability of the bonobo and chimpanzees MHC class I molecules with a reduced viral peptide set, including only viruses (*n* = 14), which are known to infect either bonobos or chimpanzees and may have influences on survival and fitness. We found no evidence for a worse or better adaptation to one of those viruses in our subsets for bonobos or chimpanzees. Obviously, the number of different viruses in this subset is quite small, however, studies working on viruses in chimpanzees and bonobos are quite rare especially studies in wild living individuals, therefore limiting the number of potential candidates.

Our study, although comprehensive, has certain limitations. For example, we classified peptides as “bound” or “unbound” and so could not take differing binding affinities into account. In addition, expression levels of the different MHC molecules may vary and thereby affect immune function [63, 64]. Furthermore, it is important to note, that our viral peptide set is only an approximate representation of the viral threats for bonobos and chimpanzees and that some viruses important for wild living bonobos and chimpanzee could be lacking in our dataset. However, the aim of this study was not to analyze individually virus specific differences between the two species, rather than analyzing the overall peptide binding ability of bonobos in comparison to chimpanzees. In this regard, the overall proportion of bound peptides appears to be low in our study. Even chimpanzee MHC molecules with the highest proportion of bound peptides of all loci in our study bound only 14.6% of viral peptides from our dataset. However, in comparison to a study analyzing the MHC class I viral peptide binding ability of humans and chimpanzees the percentage of bound peptides in our study is comparable [42]. Also the high variation of bound peptides among the different MHC molecules in our study has also been shown in human MHC class I molecules [47]. Furthermore, it is important to note that the results in this present study are limited to the classical MHC class I genes *A*, *B* and *C* which are involved in the presentation of intracellular peptides and therefore defend against viruses [4, 5]. However, alleles from MHC class I genes are also involved in the interaction with KIR receptors on NK cells [6, 7]. A study of samples from wild bonobo populations found both the Bw4 and C1 KIR receptor epitopes on bonobo MHC-*B* molecules [27]. This indicates, that even though bonobos have a reduced peptide binding ability at MHC class I genes compared to chimpanzees, they still obtain the same KIR receptor epitopes like chimpanzees at the *B* locus [65], suggesting an unrestricted interaction with NK cells in bonobos. Nevertheless, it is important to mention, that an organism's immune defense is not solely dependent on MHC genes and that a substantial proportion of non-MHC genes is also responsible for resistance to infectious diseases [66].

The bioinformatic tool, *NetMHCpan* used to predict the binding specificities in this study is based on an artificial neural network trained with MHC sequences from several species, including chimpanzees [38, 39]. Although bonobo MHC sequences were not included in the training of the artificial neural network, the close relatedness of chimpanzees and bonobos, which is also apparent on the level of MHC class I loci [26], suggests that *NetMHCpan* should not perform very differently on bonobo than on chimpanzee MHC class I molecules. The predicted performance of *NetMHCpan* can be estimated from the distance to the nearest neighbor of the training data, as a small distance represents a better performance [38, 39]. A comparison of both species using *NetMHCpan* showed only a slight difference between bonobos and chimpanzees, whereby the mean distance of bonobo MHC sequences to the training data from this study were 14.5% higher compared to the chimpanzee distance. However, the highest distance of all bonobo MHC molecules were 9 % smaller compared to chimpanzees. We thus conclude that any species-specific effects in our use of *NetMHCpan* on bonobo and chimpanzees MHC sequences would have at most a small effect that would not explain the results of our study. Four of our MHC molecules did not bind any peptides after applying the binding affinity threshold of 500 nM, indicating that this threshold was too stringent for those four molecules. However, these four molecules represent only a very small fraction (1.83 %) of the total number of MHC molecules tested in this study.

In conclusion, this study shows the usefulness of a bioinformatic tool to predict binding specificities of MHC molecules to potential bound peptides to investigate differences in the peptide binding ability of two closely related species. The analysis of peptide and MHC molecule interaction in laboratory experiments is vastly time consuming and cost intense especially, when trying to analyze large sets of peptides or MHC molecules [34]. Here we demonstrate that bioinformatic binding prediction tools are a promising alternative to laboratory experiments to investigate such large-scale questions. The comparison between bonobos and chimpanzees showed a reduced peptide binding ability of bonobos mainly at the *B* and *C* loci. This is in concordance with a reduced diversity of bonobos at these loci compared to chimpanzees [26–28]. In the light of the functional importance of MHC molecules for the immune system this could be a result of a selective process caused by certain pathogens. To what degree this reduced peptide binding ability in bonobos has an effect on the survivability and fitness on individuals of wild bonobo populations is unclear. Nevertheless, concerning their status as an endangered species, special caution is needed to prevent introduction and spread of pathogens to bonobos, as their ability to counter new pathogens could be limited.

## Additional files

Additional file 1: MHC molecules and number of predicted bound peptides (Microsoft Excel worksheet .xlsx): Table shows the names of MHC class I molecules used in this study and the predicted number of peptides for each MHC molecule. (XLSX 17 kb)

Additional file 2: Genotypes for the MHC class I *A*, *B* and *C* loci for 20 bonobos, central chimpanzees and western chimpanzees (Microsoft Excel worksheet .xlsx): Table includes genotypes for each of the 20 individual bonobos, central and western chimpanzees taken from the literature. (XLSX 14 kb)

Additional file 3: Frequency of peptide sequences (Microsoft Excel worksheet .xlsx): Table shows the frequency of an identical peptide sequence in the dataset with the corresponding number of peptides with this particular sequence. (XLSX 14 kb)

Additional file 4: Statistic results of the “population”- and “individual”-level comparison (Microsoft Excel worksheet .xlsx): Table showing the statistic results of the “population” -level and “individual”-level comparisons for the analysis of the number of bound peptides for each of the particular comparisons. (XLSX 11 kb)

## Abbreviations

KIR receptors: Killer cell immunoglobulin-like receptors
MHC: Major histocompatibility complex
mya: Million years ago
NK cells: Natural killer cells
nM: Nanomolar
SIV: Simian immunodeficiency virus

## References

[1] Codner GF, Stear MJ, Reeve R, Matthews L, Ellis SA (2012). Selective forces shaping diversity in the class I region of the major histocompatibility complex in dairy cattle. Anim Genet.

[2] Geraghty DE, Daza R, Williams LM, Vu Q, Ishitani A (2002). Genetics of the immune response: identifying immune variation within the MHC and throughout the genome. Immunol Rev.

[3] Kelley J, Walter L, Trowsdale J (2005). Comparative genomics of major histocompatibility complexes. Immunogenetics.

[4] Sommer S (2005). The importance of immune gene variability (MHC) in evolutionary ecology and conservation. Front Zool.

[5] Rock KL, Reits E, Neefjes J (2016). Present yourself! By MHC class I and MHC class II molecules. Trends Immunol.

[6] Parham P, Abi-Rached L, Matevosyan L, Moesta AK, Norman PJ, Older Aguilar AM, Guethlein LA (2010). Primate-specific regulation of natural killer cells. J Med Primatol.

[7] Parham P, Gupta S, Paul WE, Steinman R (2005). Influence of KIR diversity on human immunity. Mechanisms of lymphocyte activation and immune regulation X: innate immunity.

[8] Adams EJ, Parham P (2001). Species-specific evolution of MHC class I genes in the higher primates. Immunol Rev.

[9] Langergraber KE, Prüfer K, Rowney C, Boesch C, Crockford C, Fawcett K, Inoue E, Inoue-Muruyama M, Mitani JC, Muller MN (2012). Generation times in wild chimpanzees and gorillas suggest earlier divergence times in great ape and human evolution. Proc Natl Acad Sci.

[10] Prufer K, Munch K, Hellmann I, Akagi K, Miller JR, Walenz B, Koren S, Sutton G, Kodira C, Winer R (2012). The bonobo genome compared with the chimpanzee and human genomes. Nature.

[11] Takemoto H, Kawamoto Y, Furuichi T (2015). How did bonobos come to range south of the Congo river? Reconsideration of the divergence of Pan paniscus from other Pan populations. Evol Anthropol.

[12] Adams EJ, Cooper S, Parham P (2001). A novel, nonclassical MHC class I molecule specific to the common chimpanzee. J Immunol.

[13] Gleimer M, Wahl AR, Hickman HD, Abi-Rached L, Norman PJ, Guethlein LA, Hammond JA, Draghi M, Adams EJ, Juo S (2011). Although divergent in residues of the peptide binding site, conserved chimpanzee Patr-AL and polymorphic human HLA-A*02 have overlapping peptide-binding repertoires. J Immunol.

[14] Fischer A, Wiebe V, Paabo S, Przeworski M (2004). Evidence for a complex demographic history of chimpanzees. Mol Biol Evol.

[15] Fischer A, Pollack J, Thalmann O, Nickel B, Paabo S (2006). Demographic history and genetic differentiation in apes. Current biology : CB.

[16] Becquet C, Patterson N, Stone AC, Przeworski M, Reich D (2007). Genetic structure of chimpanzee populations. PLoS Genet.

[17] Prado-Martinez J, Sudmant PH, Kidd JM, Li H, Kelley JL, Lorente-Galdos B, Veeramah KR, Woerner AE, O'Connor TD, Santpere G (2013). Great ape genetic diversity and population history. Nature.

[18] Kawamoto Y, Takemoto H, Higuchi S, Sakamaki T, Hart JA, Hart TB, Tokuyama N, Reinartz GE, Guislain P, Dupain J (2013). Genetic structure of wild bonobo populations: diversity of mitochondrial DNA and geographical distribution. PLoS One.

[19] Eriksson J, Hohmann G, Boesch C, Vigilant L (2004). Rivers influence the population genetic structure of bonobos (Pan paniscus). Mol Ecol.

[20] Slade RW, McCallum HI, Vs O (1992). Frequency-dependent selection at Mhc loci. Genetics.

[21] Penn DJ, Potts WK (1999). Associate editor: Stephen RP: the evolution of mating preferences and major histocompatibility complex genes. Am Nat.

[22] Bernatchez L, Landry C (2003). MHC studies in nonmodel vertebrates: what have we learned about natural selection in 15 years?. J Evol Biol.

[23] Spurgin LG, Richardson DS (2010). How pathogens drive genetic diversity: MHC, mechanisms and misunderstandings. Proc R Soc B Biol Sci.

[24] Sidney J, Grey HM, Kubo RT, Sette A (1996). Practical, biochemical and evolutionary implications of the discovery of HLA class I supermotifs. Immunol Today.

[25] Lighten J, Papadopulos AST, Mohammed RS, Ward BJ, G. Paterson I, Baillie L, Bradbury IR, Hendry AP, Bentzen P, van Oosterhout C: Evolutionary genetics of immunological supertypes reveals two faces of the red queen. Nat Commun 2017, 8(1):1294. 10.1038/s41467-017-01183-2 PMC5670221 29101318

[26] Maibach V, Hans JB, Hvilsom C, Marques-Bonet T, Vigilant L (2017). MHC class I diversity in chimpanzees and bonobos. Immunogenetics.

[27] Wroblewski EE, Guethlein LA, Norman PJ, Li Y, Shaw CM, Han AS, Ndjango J-BN, Ahuka-Mundeke S, Georgiev AV, Peeters M, et al. Bonobos maintain immune system diversity with three functional types of MHC-B. J Immunol. 2017. 10.4049/jimmunol.1601955 PMC5469624 28348269

[28] de Groot NG, Heijmans CMC, Helsen P, Otting N, Pereboom Z, Stevens JMG, Bontrop RE. Limited MHC class I intron 2 repertoire variation in bonobos. Immunogenetics. 2017;69(10):677–88. 10.1007/s00251-017-1010-x 28623393

[29] Meyer-Lucht Y, Sommer S (2005). MHC diversity and the association to nematode parasitism in the yellow-necked mouse (Apodemus flavicollis). Mol Ecol.

[30] Radwan J, Biedrzycka A, Babik W (2010). Does reduced MHC diversity decrease viability of vertebrate populations?. Biol Conserv.

[31] Aguilar A, Roemer G, Debenham S, Binns M, Garcelon D, Wayne RK (2004). High MHC diversity maintained by balancing selection in an otherwise genetically monomorphic mammal. Proc Natl Acad Sci U S A.

[32] Radwan J, Zagalska-Neubauer M, CichoŃ M, Sendecka J, Kulma K, Gustafsson L, Babik W (2012). MHC diversity, malaria and lifetime reproductive success in collared flycatchers. Mol Ecol.

[33] Castro-Prieto A, Wachter B, Sommer S (2011). Cheetah paradigm revisited: MHC diversity in the World's largest free-ranging population. Mol Biol Evol.

[34] Lundegaard C, Lund O, Buus S, Nielsen M (2010). Major histocompatibility complex class I binding predictions as a tool in epitope discovery. Immunology.

[35] Pro SC, Zimic M, Nielsen M (2014). Improved pan-specific MHC class I peptide binding predictions using a novel representation of the MHC binding cleft environment. Tissue Antigens.

[36] Robinson J, Halliwell JA, McWilliam H, Lopez R, Marsh SGE (2013). IPD—the Immuno polymorphism database. Nucleic Acids Res.

[37] Vita R, Overton JA, Greenbaum JA, Ponomarenko J, Clark JD, Cantrell JR, Wheeler DK, Gabbard JL, Hix D, Sette A (2015). The immune epitope database (IEDB) 3.0. Nucleic Acids Res.

[38] Nielsen M, Lundegaard C, Blicher T, Lamberth K, Harndahl M, Justesen S, Røder G, Peters B, Sette A, Lund O (2007). NetMHCpan, a method for quantitative predictions of peptide binding to any HLA-A and -B locus protein of known sequence. PLoS One.

[39] Hoof I, Peters B, Sidney J, Pedersen LE, Sette A, Lund O. NetMHCpan, a method for MHC class I binding prediction beyond humans. Immunogenetics. 2009;61. 10.1007/s00251-008-0341-z PMC3319061 19002680

[40] Nielsen M, Andreatta M (2016). NetMHCpan-3.0; improved prediction of binding to MHC class I molecules integrating information from multiple receptor and peptide length datasets. Genome Medicine.

[41] Mihara T, Nishimura Y, Shimizu Y, Nishiyama H, Yoshikawa G, Uehara H, Hingamp P, Goto S, Ogata H. Linking virus genomes with host taxonomy. Viruses. 2016;8(3). 10.3390/v8030066 PMC4810256 26938550

[42] van Deutekom HW, Hoof I, Bontrop RE, Kesmir C (2011). A comparative analysis of viral peptides presented by contemporary human and chimpanzee MHC class I molecules. J Immunol.

[43] de Groot NG, Otting N, Robinson J, Blancher A, Lafont BAP, Marsh SGE, O’Connor DH, Shiina T, Walter L, Watkins DI (2012). Nomenclature report on the major histocompatibility complex genes and alleles of great ape, old and New World monkey species. Immunogenetics.

[44] Brister JR, Ako-Adjei D, Bao Y, Blinkova O (2015). NCBI viral genomes resource. Nucleic Acids Res.

[45] Kanz C, Aldebert P, Althorpe N, Baker W, Baldwin A, Bates K, Browne P, van den Broek A, Castro M, Cochrane G et al: The EMBL nucleotide sequence database. Nucleic Acids Res 2005, 33(Database Issue):D29-D33. 10.1093/nar/gki098 PMC540052 15608199

[46] Sette A, Vitiello A, Reherman B, Fowler P, Nayersina R, Kast WM, Melief CJ, Oseroff C, Yuan L, Ruppert J (1994). The relationship between class I binding affinity and immunogenicity of potential cytotoxic T cell epitopes. J Immunol.

[47] Paul S, Weiskopf D, Angelo MA, Sidney J, Peters B, Sette A (2013). HLA class I alleles are associated with peptide-binding repertoires of different size, affinity, and immunogenicity. J Immunol.

[48] Thomsen M, Lundegaard C, Buus S, Lund O, Nielsen M (2013). MHCcluster, a method for functional clustering of MHC molecules. Immunogenetics.

[49] Adams EJ, Cooper S, Thomson G, Parham P (2000). Common chimpanzees have greater diversity than humans at two of the three highly polymorphic MHC class I genes. Immunogenetics.

[50] Adams DC, Anthony CD (1996). Using randomization techniques to analyse behavioural data. Anim Behav.

[51] Groves CP (2001). Primate taxonomy.

[52] de Manuel M, Kuhlwilm M, Frandsen P, Sousa VC, Desai T, Prado-Martinez J, Hernandez-Rodriguez J, Dupanloup I, Lao O, Hallast P (2016). Chimpanzee genomic diversity reveals ancient admixture with bonobos. Science.

[53] Sitbon E, Pietrokovski S (2007). Occurrence of protein structure elements in conserved sequence regions. BMC Struct Biol.

[54] Nei M, Rooney AP (2005). Concerted and birth-and-death evolution of multigene families. Annu Rev Genet.

[55] Nei M, Gu X, Sitnikova T (1997). Evolution by the birth-and-death process in multigene families of the vertebrate immune system. Proc Natl Acad Sci U S A.

[56] Goyos A, Guethlein LA, Horowitz A, Hilton HG, Gleimer M, Brodsky FM, Parham P (2015). A distinctive cytoplasmic tail contributes to low surface expression and intracellular retention of the Patr-AL MHC class I molecule. J Immunol.

[57] de Groot NG, Stevens JMG, Bontrop RE. Does the MHC confer protection against malaria in bonobos? Trends Immunol. 2018. 10.1016/j.it.2018.07.004 30126696

[58] de Groot NG, Otting N, Doxiadis GGM, Balla-Jhagjhoorsingh SS, Heeney JL, van Rood JJ, Gagneux P, Bontrop RE (2002). Evidence for an ancient selective sweep in the MHC class I gene repertoire of chimpanzees. Proc Natl Acad Sci U S A.

[59] de Groot NG, Heijmans CMC, De Groot N, Otting NEL, De Vos-Rouweler AJM, Remarque EJ, Bonhomme M, Doxiadis GGM, Crouau-Roy B, Bontrop RE (2008). Pinpointing a selective sweep to the chimpanzee MHC class I region by comparative genomics. Mol Ecol.

[60] de Groot NG, Bontrop RE (2013). The HIV-1 pandemic: does the selective sweep in chimpanzees mirror humankind’s future?. Retrovirology.

[61] de Groot NG, Heijmans CMC, Bontrop RE (2017). AIDS in chimpanzees: the role of MHC genes. Immunogenetics.

[62] Sharp PM, Hahn BH (2011). Origins of HIV and the AIDS pandemic. Cold Spring Harbor perspectives in medicine.

[63] Parolini F, Biswas P, Serena M, Sironi F, Muraro V, Guizzardi E, Cazzoletti L, Scupoli MT, Gibellini D, Ugolotti E, et al. Stability and Expression Levels of HLA-C on the Cell Membrane Modulate HIV-1 Infectivity. J. Virol. 2017;92(1):e01711-01717. 10.1128/JVI.01711-17 PMC5730790 29070683

[64] Shi B, Thomas AJ, Benninghoff AD, Sessions BR, Meng Q, Parasar P, Rutigliano HM, White KL, Davies CJ (2018). Genetic and epigenetic regulation of major histocompatibility complex class I gene expression in bovine trophoblast cells. Am J Reprod Immunol.

[65] Parham P, Moffett A (2013). Variable NK cell receptors and their MHC class I ligands in immunity, reproduction and human evolution. Nat Rev Immunol.

[66] Acevedo-Whitehouse K, Cunningham AA (2006). Is MHC enough for understanding wildlife immunogenetics?. Trends Ecol Evol.

